# Laryngectomies partielles supra-cricoïdiennes avec reconstruction par CHEP: notre expérience sur 16 cas

**DOI:** 10.11604/pamj.2017.27.191.11955

**Published:** 2017-07-13

**Authors:** Bouchaib Hemmaoui, Mohamed Sahli, Noureddine Errami, Ahmed Rouihi, Mohamed Habib Bahalou, Ilias Benchaifai, Amine Ennouali, Sara Britel, Ismail Nakkabi, Ali Jahidi, Mohamed Zalagh, Saloua Ouaraini, Fouad Benariba

**Affiliations:** 1 Service d’ORL et Chirurgie Cervico-faciale, Hôpital d’Instruction Militaire Mohamed V, Rabat, Maroc

**Keywords:** Chirurgie partielle, crico-hyoïdo-épiglottopexie, complications, contrôle carcinologique, Partial surgery, crico-hyoido-epiglottopexy, complications, carcinologic control

## Abstract

La chirurgie partielle du larynx avec reconstruction par crico-hyoïdo-épiglottopexie (CHEP) s’adresse essentiellement aux cancers du plan glottique permettant une préservation satisfaisante des rôles physiologiques et un contrôle carcinologique local également satisfaisant. L’objectif de notre étude est d’analyser les résultats fonctionnels et carcinologiques de cette chirurgie. Il s’agit d’une étude rétrospective incluant l’ensemble des patients ayant bénéficié d’une chirurgie partielle du larynx avec reconstruction CHEP entre 2011 et 2014 dans notre institution. Nous avons analysé les données épidémiologiques, les particularités chirurgicales, les suites fonctionnels et le contrôle carcinologique de la maladie. Au total, 16 patients ont été inclus dans cette étude. Tous nos patients avaient un carcinome épidermoïde du plan glottique classé T1 ou T2. Les suites fonctionnels étaient généralement simples surtout dans les cas où La conservation des 2 unités cricoaryténoïdiennes était possible (75% des cas) mais avec toutefois des complications post-opératoires notées dans 31,25%. Le contrôle carcinologique était satisfaisant, une seule récidive locale a été objectivée dans notre étude. La chirurgie partielle du larynx avec reconstruction par crico-hyoïdo-épiglottopexie (CHEP) est une chirurgie sure avec une préservation des rôles physiologiques et une qualité de vie satisfaisante, elle permet également un bon contrôle carcinologique sous réserve de bien cerner les indications.

## Abstract (en)

Partial laryngectomy with either cricohyoidoepiglottopexy (CHEP) are mainly used to treat glottic cancers becuse they ensure a satisfactory preservation of physiological functions and satisfactory local carcinologic control. Our study aimed to analyze the functional and carcinologic results of this surgical technique. We conducted a retrospective study of patients undergoing partial laryngectomy with either cricohyoidoepiglottopexy in our Hospital between 2011 and 2014. We analyzed the epidemiological data, the surgical peculiarities, the functional outcomes and the carcinologic control of the disease. A total of 16 patients were included in this study. All our patients had T1 or T2 glottis squamous cell carcinoma. Functional outcomes were generally simple, especially in cases where the preservation of the 2 cricoarytenoid units was possible (75% of cases). However post-operative complications were reported in 31.25%. Carcinologic control was satisfactory, only one patient experienced local recurrence. Partial laryngectomy with either cricohyoidoepiglottopexy (CHEP) is a safe surgery preserving physiological functions and ensuring satisfactory quality of life. It also allows for good carcinologic control (it is subject of course to compliance with surgical indications).

## Introduction

Le cancer du larynx représente 3,5% des tumeurs malignes diagnostiquées annuellement au monde et il est à l’origine d’environ 1% de décès par cancer [1]. Plusieurs modalités thérapeutiques sont décrites avec des indications précises pour chacune et avec pour objectif la guérison et lapréservation de l’intégrité physique et psychologique dupatient, chacune de ses modalités a ses avantages mais avec un contrôle carcinologique local comparable. Notre travail s’intéresse rétrospectivement à une série de patients ayant un carcinome épidermoïde du plan glottique, dont la décision thérapeutique a été une chirurgie partielle. Le but decette étude est d’évaluer les résultats carcinologiques et fonctionnels de cette chirurgie partielle.

## Méthodes

Il s’agit d’une étude rétrospective sur 4 ans (de 2011 à 2014) incluant 16 patients ayant bénéficié d’une chirurgie partielle supra-cricoïdienne avec reconstruction par crico-hyoïdo-épiglottopexie (LPSC-CHEP) pour un carcinome épidermoïde du plan glottique classe T1 ou T2. L’étude a porté sur les données épidémiologiques, le stade de la lésion primitive, les particularités chirurgicales, les complications post opératoires et l’analyse du contrôle de la maladie. Tous nos patients ont bénéficié d’un bilan endoscopique et radiologique et ne présentaient pas de contre-indication à une chirurgie laryngée partielle. Les indications opératoires ont été discutées en réunion de concertation pluridisciplinaire. Un traitement chirurgical sur les aires ganglionnaires a été réalisé de façon concomitante au geste laryngé chez 2 patients qui étaient classés N1.

## Résultats

**Figure 1:**
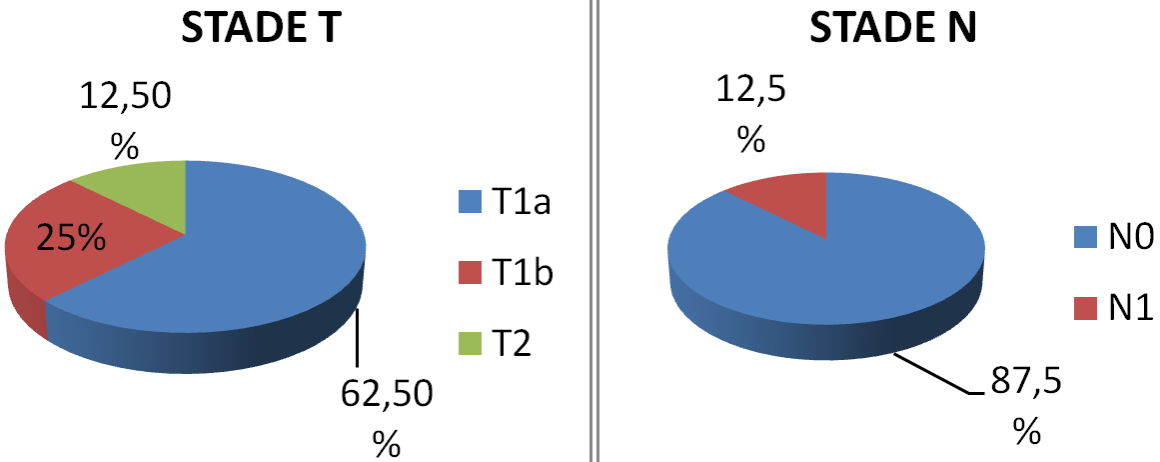
Classification TNM de nos patients

**Figure 2:**
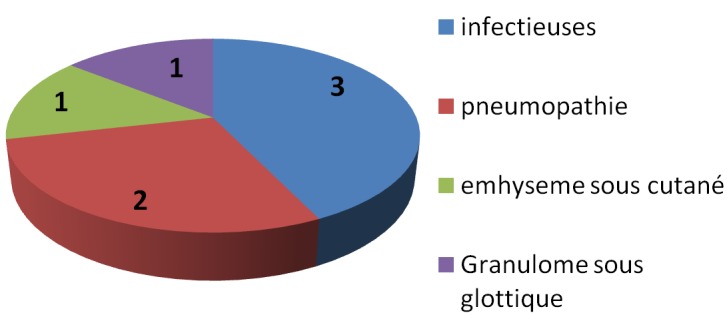
Complications postopératoires notées dans notre série

L’âge moyen de nos patients était de 53 ans, tous nos patients étaient de sexe masculin. Le facteur de risque principal était le tabac (93,75% des patients). Les patients ont été suivis pour une durée moyenne de deux ans et demi après leurs chirurgies (± 1,9 ans). La classification TNM de nos patients est représentée dans la Figure 1: 62,5 % des patients étaient classes T1a, 25% étaient classés T1b et 12,5 % classés T2. Pour le stade N, 87,5% des patients étaient classés N0, et 12,5 % étaient classés N1. Cent pour cent des patients étaient classés M0. La chirurgie s’est déroulée selon la technique classique décrite par Piquet. La conservation des 2 unités cricoaryténoïdiennes a été faite dans 75% des cas (cas où le contrôle carcinologique était évident). Sur le plan histologique, un carcinome épidermoïde était noté chez tous nos patients. Les limites étaient également saines, sauf un seul patient qui avait une infiltration sous muqueuse non visible macroscopiquement chez qui une laryngectomie totale de rattrapage a été faite. Un seul patient avait une atteinte du ganglion Delphien chez qui une radiothérapie post opératoire a été faite. Les complications postopératoires immédiates notées dans 5 cas (soit 31,25%) sont illustrées dans la Figure 2. Les complications infectieuses rapportées dans 18,75% des cas étaient en rapport avec une infection de l’orifice de trachéotomie apparue après un délai moyen de 12 jours. Ces patients ont bénéficié d’un prélèvement bactériologique par écouvillonnage et d’une antibiothérapie adaptée (deux cas de *Pseudomonas aeruginosa* et un cas de pneumocoque Sensible à la ciprofloxacine). L’évolution était favorable par la suite. D’autres complications postopératoires ont été notées: 2 cas de pneumopathie d’inhalation (12,5% des cas) avec un délai moyen de 8j, un cas d’emphysème sous cutané minime ayant régressé spontanément et un cas de granulome sous glottique ayant bénéficié d’une exérèse endoscopique. Sur le plan fonctionnel, le délai moyen de décanulation était de 10 jours (7-12j) et les premiers essais alimentaires ont commencé entre le 8^ème^ et le 15^ème^ jour post-opératoire. La durée d’utilisation de la sonde de gastrostomie était en moyenne de 15jours. Tous nos patients ont bénéficié d’une rééducation orthophonique qui a débuté quelques jours après l’intervention avec une bonne qualité de voix par la suite. Sur le plan carcinologique, on dénombre un seul échec local dans un contexte poly-métastatique (poumon, os, cerveau, moelle cervicale), pour lequel une radiothérapie stéréotaxique et une chimiothérapie palliative a été faite. Ce patient avait des facteurs prédictifs de mauvais pronostic (caractère peu différencié, engainement péri nerveux). La radiothérapie n’était pas systématique, sauf pour un patient qui etait atteinte du ganglion Delphien. Aucune récidive locale n’a été observée pour 62,5 % des patients avec un recul moyen de 3 ans.

## Discussion

La prise en charge de cancers du larynx bénéficie actuellement d’un progrès considérable en matière d’exploration et de traitement, dont l’objectif final est de conserver au maximum les fonctions physiologiques. Certes le protocole de radiothérapie/chimiothérapie a des résultats fonctionnels souvent considérés comme meilleurs mais les résultats sont comparables avec ceux de la chirurgie partielle du larynx de point de vue carcinologique [2]. Cette chirurgie garde toutefois ses avantages en évitant une chirurgie de rattrapage sur terrain irradié dont les suites fonctionnels sont plus lourdes [3]. LPS-CHEP est une chirurgie conservatrice du larynx indiquée non seulement pour traiter les cancers au stade précoce ou intermédiaire mais également certains cancersavancés bien sélectionnés en permettant une résection d’environ trois quarts du larynx y compris les espaces paraglottiques [4]. Les objectifs de cette chirurgie sont: (i) un bon contrôle oncologique post opératoire, (ii) éviter une trachéostomie (iii) préserver des fonctions vocales et de déglutition acceptables. Cette chirurgie s’adresse aux tumeurs classées T1, T2 et certains T3 [5]: par atteinte du plan glottique avec une mobilité conservée; par atteinte de l’espace paraglottique et/ou atteinte du périchondre interne du cartilage thyroïde; sans atteinte de l’espace pré-épiglottique et sans extension sous glottique (une extension tumorale de 5 mm sous le plan glottique peut être admise). Le curage ganglionnaire n’est pas préconisé pour les tumeurs classées T1 car elles ne donnent pas de métastases ganglionnaires. Mais pour les tumeurs classées T2-T3 glottiques, un curage des aires II, III et IV homolatéral est généralement réalisé [5], ce qui était le cas de 2 patients de notre série. Des variantes chirurgicales ont été décrites, notamment la conservation des 2 unités cricoaryténoïdiennes (si elle est possible carcinologiquement) qui raccourcit les suites fonctionnelles. Cette conservation était possible dans 75% de nos malades. Ceci étant dû en premier au stade précoce de découverte du cancer glottique. La décanulation précoce facilite l’ascension du larynx et la reprise de la déglutition. Le délai moyen de décanulation dans notre série était de 10 jours, celui rapporté dans littérature variait de 9 à 30jours. Les facteurs prédictifs rapportés dans littérature d’un retard de décanulation sont l’âge avancé et l’œdème aryténoïdien post opératoire [5]. Le délai de l’ablation de la sonde nasogastrique est très variable dans littérature allant de 15 jours jusqu’à à 90jours avec en général un taux d’ablation de la sonde de 90% durant le premier mois postopératoire [6]. Dans notre série le délai moyen d’ablation de la SNG ou la gastrostomie était de 23j (15 à 90j). En général, les résultats fonctionnels sont différents d’une série à l’autre pouvant être en rapport avec des populations différentes et des habitudes de l’équipe. En effet dans notre expérience, nous avons préconisé d’utiliser plus la sonde de gastrostomie que la SNG car elle permet d’éviter le problème du reflux gastro œsophagien et l’œdème aryténoïdien qui gêne la décanulation précoce. L’ensemble de ces résultats fonctionnels sont résumés dans le Tableau 1 [5–7].

**Tableau 1:** Résultats fonctionnels de la LPS-CHEP dans différentes séries

Auteur/Année	Nombre de cas	Délai de la SNG	Délai de décanulation	Alimentation orale (%)
Naudo et al 1997 [5]	122	22j	8j (3-90j)	-
De Vincentiis et al 1998 [7]	51	15j	25j (15-90j)	-
Bron et al 2000 [6]	69	22j	27j (8-85j)	-
Notre étude				

La chirurgie partielle du larynx est considéré comme une chirurgie propre contaminée (exposition de l’axe aérodigestif), d’autant plus qu’elle implique souvent en post-opératoire des matériaux étrangers (drains, SNG, canule de trachéotomie). La durée de l’antibioprophylaxie pour cette chirurgie propre contaminée est en principe inférieure à 48h.Cependant, plusieurs études plaident pour une antibiothérapie prolongée vu le pourcentage plus important de complications infectieuses post opératoires. Ce taux était d’environ 18,75% dans notre étude. Les facteurs prédictifs les plus rapportés dans la littérature sont [8]: l’extension tumorale et ganglionnaire, les évidements bilatéraux radicaux, les facteurs locaux et généraux notamment le diabète, les facteurs post-opératoires surtout l’hématome. Les complications respiratoires sont assez fréquentes dans ce type de chirurgie, en effet l’incidence de ces complications est variables d’une série à l’autre pouvant aller de 1, 2 à 38% [6]. Le délai de décanulation est le facteur le plus déterminant pour la réussite fonctionnelle en termes de respiration, et son retard peut être associé à une sténose laryngée, un granulome et un œdème aryténoïdien.L’éviction de l’inhalation de la saliveen post opératoire immédiat doit être strictement effectuée pour éviter une pneumopathie d’inhalation post-opératoire et ceci doit être bien expliqué au patient avant l’intervention chirurgicale. L’incidence de la pneumonie d'inhalation après LPS-CHEP varie de 0% à 9% dans la littérature européenne [9]. La conservation d’une unité crico aryténoïdienne est également décrite dans la littérature comme un facteur important dans les suites fonctionnelles, mais des études ciblées réalisées dans ce sens n’ont pas objectivé de différence significative en termes de suites fonctionnelles [10, 11]. D’autres complications ont été rapportés dans la littérature et peuvent être responsable d’une dyspnée inspiratoire et sont en rapport entre autre soit avec avec un retard de décanulation ou des erreurs techniques [5, 12]: un granulome, un œdème, une sténose laryngé, un emphysème sous-cutané extensif, une laryngocèle ou une rupture de pexie. La laryngocèle est considérée comme erreur technique car elle est liée à la section du toit du ventricule Morgagni, au moment de la laryngotomie trans-epiglottique. La rupture de péxie quant à elle, est une complication très rare et doit être évitée par une dissection le long de la face antérieure de la trachée pour faciliter l’affrontement des berges de la pexie. Dans notre série, nous avons noté un seul cas de granulome infra-glottique et un seul cas d’emphysème sous cutané. Le contrôle carcinologique est satisfaisant dans la plupart des études quoique les résultats soient difficiles à comparer dépendant essentiellement du stade et de la localisation tumorale, en effet la localisation sus glottique est de mauvais pronostic que la localisation glottique (survie globale de 66% à trois ans contre 80 à 88% pour les localisations glottiques). D’autres études montrent que les résultats dépendent également du terrain: en territoire non irradié le contrôle local est de 90% et le taux de survie est de 80% à 5ans; alors que en territoire irradiée le taux de contrôle chute jusqu’à 61% et le taux de survies de 66 à 85% à 3 ans [13, 14]. Dans notre série le contrôle local était de 93,75% avec un seul échec local ganglionnaire. Le caractère peu différencié, et la présence d’engainementspérinerveux sont les facteurs pronostics les plus rapportés dans la littérature [15]. Ces éléments prédictifs ont été rapportés à l’examen anatomopathologique initial chez ce patient expliquant l’évolution et l’agressivité de sa maladie. L’envahissement tumoral du « ganglion Delphien » est un autre élément pronostic majeur qui influait de façon statistiquement significative sur l’apparition secondaire de métastase lymphonodale cervicale et sur l’apparition de récidive tumorale locale (Tableau 2) [16–18].

**Tableau 2:** Résultats oncologiques de la LPS-CHEP dans différentes séries

Leone et al [16]	152 cas	92,1%	83,5%
Park et al [17]	116 cas	89,7%	66,6%
Laccourreye et al [18]	62 cas	98,2%	86,5%
Notre série	16 cas	93,75%	-

## Conclusion

La chirurgie partielle du larynx est une chirurgie attirante pour les ORL et elle est considéré comme une stratégie sure pour la conservation d’organe avec un contrôle local très satisfaisant dans la plupart des séries. Elle permet en outre une conservation des 3 fonctions physiologiques principales de déglutition, respiration et de phonation. Cependant, les suites post opératoires sont parfois longues avec des complications fréquentes, d’où l’intérêt d’une prise en charge correcte et à temps.

### Etat des connaissances actuelle sur le sujet

- La chirurgie partielle du larynx avec reconstruction par crico-hyoïdo-épiglottopexie (CHEP) s’adresse essentiellement aux cancers du plan glottique;
- C’est chirurgie avec une préservation satisfaisante des rôles physiologiques du larynx.

### Contribution de notre étude à la connaissance

- Notre étude est basée sur l’analyser des résultats fonctionnels et carcinologiques de cette chirurgie partielle;
- Une prise en charge adéquate des complications post opératoire permet au patient une cicatrisation rapide, décanulation précoce et reprise des ses fonctions précocement.

## Conflits d’intérêts

Les auteurs ne déclarent aucun conflit d'intérêt.
